# Bioavailability of Sodium Iron Ethylenediaminetetraacetate, Ferrous Fumarate, and Ferrous Sulfate in Corn Flour Using Different INFOGEST Digestion and Caco-2 Cell Model Methods

**DOI:** 10.1016/j.cdnut.2026.107686

**Published:** 2026-04-03

**Authors:** Jiejia Zhang, Brian L Lindshield

**Affiliations:** School of Health Sciences, College of Human Health and Sciences, Kansas State University, Manhattan, KS, United States

**Keywords:** INFOGEST, Caco-2 cell model, dialysis membrane, insert membrane, digesta, supernatant, iron bioavailability

## Abstract

**Background:**

The static digestion/Caco-2 cell model has been widely employed as a bioassay to assess iron bioavailability. However, there are many methodological differences in how it is utilized. Thus, the feasibility of different chamber systems and iron treatment options was examined.

**Objectives:**

To investigate iron bioavailability of INFOGEST digesta compared with supernatant in Caco-2 model with Glahn’s dual-chamber system (dialysis membrane), an alternative dual-chamber system (insert membrane), or a triple-chamber system (insert membrane + dialysis membrane). To determine the bioavailability of precooked corn flour fortified with sodium iron ethylenediaminetetraacetate (NaFeEDTA), ferrous fumarate, ferrous sulfate (FeSO_4_), and their combinations.

**Methods:**

Precooked corn flour fortified with NaFeEDTA, ferrous fumarate, or FeSO_4_ were digested following the INFOGEST method. The iron solubility of supernatant was analyzed, and Caco-2 cells were treated with iron digesta or supernatant in the dual-chamber or the triple-chamber systems for 2 h, respectively. The ferritin concentrations were quantified to compare systems. Finally, the bioavailability of NaFeEDTA, ferrous fumarate, and FeSO_4_ combinations was measured using Glahn’s dual-chamber system.

**Results:**

Iron solubility of NaFeEDTA was significantly higher than ferrous fumarate and FeSO_4_. The combinations of NaFeEDTA and fumarate/FeSO_4_ at the 4:9 weight ratio had significantly higher iron solubility than combinations of NaFeEDTA and fumarate/FeSO_4_ at the 2:11 weight ratio. Ferritin concentrations in Glahn’s dual-chamber system were significantly higher than the alternative dual-chamber and the triple-chamber systems. The combination of NaFeEDTA and FeSO_4_ at a 2:11 weight ratio had significantly higher ferritin concentrations than the other combination samples.

**Conclusions:**

The Caco-2 cell model with Glahn’s dual-chamber system and INFOGEST digesta was the best treatment and chamber system. NaFeEDTA had significantly higher bioavailability than FeSO_4_, but not ferrous fumarate. The combination of NaFeEDTA and FeSO_4_ at a 2:11 weight ratio was the most promising iron treatment ratio.

## Introduction

*In vitro* iron bioassays are constantly developed, refined, and advanced as prediction tools. In theory, *in vitro* iron solubility should be a good predictor of *in vivo* iron absorption and bioavailability because the more solubilized iron, the more iron can be absorbed and utilized [1]. However, that has not always proven to be the case when investigated [[2], [3], [4]]. Processed complementary foods contained significantly more soluble iron than unprocessed complementary foods [2]. However, the subsequent clinical trial revealed no significant difference in iron absorption between the processed and unprocessed complementary food [2]. There was also no significant difference in hemoglobin concentrations between the infants fed with processed complementary foods compared with unprocessed complementary foods [5]. In Glahn’s Caco-2 cell model, soluble iron was a poor predictor of cellular iron bioavailability [4].

Previously, it was reported that the iron solubility of ferric pyrophosphate (FePP) + zinc was significantly higher than that of FePP + zinc sulfate (ZnSO_4_), but there was no difference between them in isotopic iron absorption rate. In contrast, the iron absorption of FePP + zinc oxide was significantly lower than that of FePP + ZnSO_4_, but there was no difference in their iron solubilities [6]. Further, the iron solubility of ferrous sulfate (FeSO_4_) was twice that of FePP + citric acid + trisodium citrate, but there was no difference in their iron absorption [7]. Another study comparing FePP + EDTA + zinc oxide, FePP + EDTA + ZnSO_4_, and FePP + methylenediacrylamide + ZnSO_4_ significantly improved the iron solubility, but it did not improve iron absorption [8].

In preparing for this work, methodological differences were noted in how Glahn’s model [4], or similar methods, were utilized outside the original laboratory, making it difficult to compare results. Glahn’s model mimics the physiological process of digestion and iron bioavailability in intestinal epithelial cells. In particular, a dialysis membrane is employed to reproduce the functional role of the mucin layer [4]. The standardized in vitro static digestion method, INFOGEST, can be utilized to determine iron solubility. It simulates oral cavity and gastrointestinal tract digestion with the use of electrolytes and digestive enzymes [9]. This in vitro digestion method has several advantages, including the use of amylase, lipase, defined enzyme activities, a broader range of electrolytes, and a heat-shock treatment (Supplementary Table 1). INFOGEST can be paired with the Caco-2 cell model to examine how the iron solubility of samples correlates with the iron bioavailability. INFOGEST represents methodological refinement rather than a fundamentally new approach, with key strengths including standardized electrolyte composition, enzyme activities, and digestion conditions [9]. Although studies combining INFOGEST digestion with Caco-2 cell models for assessing iron bioavailability remain relatively limited [[10], [11], [12], [13], [14]], numerous studies have applied INFOGEST protocol to evaluate other nutrients, such as proteins, bioactive peptides, lipids, starch, lactose, vitamins, minerals, and phenolic compounds, showing it has been widely adopted and utilized.

Therefore, the INFOGEST method [9] coupled with Glahn’s Caco-2 cell model [4] was adopted for the present study. It included Glahn’s dual-chamber system using an insert frame and dialysis membrane to evaluate cellular iron bioavailability. However, with the development of experimental materials, insert frames without membranes are no longer sold. Additionally, in Glahn’s model, 1.5 mL of digesta is added to the upper chamber. Glahn’s model digest concentration is 0.5‒1 g food per 15‒17 mL digestion solution (0.029‒0.067 g/mL), whereas INFOGEST’s digesta concentration is 5 g food per 35 mL digestion solution (0.143 g/mL). It is not clear if the higher INFOGEST digesta concentration would be effective in this system. Therefore, we designed 2 alternative Caco-2 cell culture systems based on Glahn’s model to test whether they could be employed. Theoretically, the dialysis membrane mimics the mucin layer so that the low molar weight iron can be absorbed [15]. This mucin layer can be expanded to generate a mucus layer [[16], [17], [18], [19]]. The molecular weight of outer mucus layer is 440 ± 50 nm when the ratio of Caco-2 cells and HT29 cells was 9:1 [[20], [21], [22], [23]]. The permeability of the inner mucus/mucin layer is around 17 kDa [15]. Thus, the 15 kDa (1‒2 nm) dialysis membrane is used in Glahn’s dual-chamber system to simulate this mucin layer [15,24]. The larger insert membrane (0.4 μm) was utilized in the alternative systems to determine whether it could be effective before it was cut out or add value when used with the dialysis membrane.

Sodium iron ethylenediaminetetraacetate (NaFeEDTA) is an iron-EDTA chelate. During digestion, a portion of the iron from NaFeEDTA can exchange with the iron in the food matrix. This exchange allows EDTA to partially protect both added and native non-heme iron from inhibitors, keeping the iron in a more absorbable form [25,26]. Ferrous fumarate and FeSO_4_ are ferrous complexes (2O^‒^‒Fe^2+^). Compared with ferric complexes, they are easily hydrolyzed in stomach and absorbed in the duodenum. However, the amount of NaFeEDTA is restricted by the WHO for children <5 y old with the daily tolerable intake amount of 1.9 mg of NaFeEDTA per kilogram of body weight. Therefore, combining NaFeEDTA with ferrous fumarate or FeSO_4_ at a ratio of 4:9 or 2:11 was recommended by the Food Aid Quality Review Report [27,28].

Corn flour is a major staple for many populations [29]. Although corn flour contains inhibitors of iron absorption like phytic acid, precooking techniques can reduce the concentrations of iron inhibitors. Thus, industrially precooked corn flour is a relevant food matrix for investigating iron fortification [29]. Although the findings are matrix-specific, the underlying mechanisms regarding iron bioavailability are not limited to corn flour.

This study was split into 2 experiments. Experiment 1 investigated whether INFOGEST digesta could be used with the dialysis membrane, identified the optimal iron treatment method for Caco-2 cell model, and determined the bioavailability of NaFeEDTA, ferrous fumarate, and FeSO_4._ In experiment 2, the optimal iron treatment method was used to measure the iron bioavailability of fortificant combinations (NaFeEDTA + fumarate/FeSO_4_).

## Methods

### Materials

NaFeEDTA and FeSO4 were purchased from Sigma Aldrich. Ferrous fumarate and inserts (Greiner Bio-One 0.4 *μ*m, 657640) were obtained from Fisher Scientific. The user manual indicates that the insert membrane can be cut out. Silicone O-rings were supplied by Uxcell (a18111400ux0199) and are made of 100% silica gel without butyl rubber. Corn flour was produced by International Grains and Cereal Limited Liability Company. All remaining materials (e.g., human salivary α-amylase, A3176, Sigma Aldrich; ferritin ELISA assay kit, FRR31-K01, Eagle Biosciences) were procured in accordance with INFOGEST protocol [9] and Glahn’s Caco-2 cell culture method [4].

### Sample preparation

The food matrix was a commercial corn product (precooked corn flour). The product nutrition label showed that every 100 g of corn flour contained 6.67 g of dietary fiber, 6.67 g of protein, 4.67 mg of iron, 273.33 mg of potassium, 0.67 mg of thiamin, 3.67 mg of niacin, 0.33 mg of riboflavin, and 163.33 μg of folic acid. In experiment 1, corn flour samples were fortified with NaFeEDTA, ferrous fumarate, and FeSO_4_. In experiment 2, corn flour samples were fortified with the combinations of NaFeEDTA and fumarate/FeSO_4_ at high/low weight ratios (4:9 or 2:11, Table 1). The recommended iron fortification concentration for the final samples, in accordance with the Food Aid Quality Review Report (United States Agency for International Development) was 130 mg/kg corn flour [27].

**TABLE 1:** Iron compounds and fortification levels used in the study.

Sample identification	Fe component	Target Fe fortification level (mg/kg food)	Fe compound concentration (mg/kg food)	Analytical Fe concentration (mg/kg food)
1	Fumarate	130	240.0	138.0
2	NaFeEDTA	130	530.0	121.4
3	FeSO_4_·7H_2_O	130	390.0	129.6
4	NaFeEDTA	40	240.0	130.6
	Fumarate	90	190.0	—
5	NaFeEDTA	40	198.6	131.3
	FeSO_4_·7H_2_O	90	270.0	—
6	NaFeEDTA	20	99.3	133.4
	Fumarate	110	191.3	—
7	NaFeEDTA	20	99.3	118.6
	FeSO_4_·7H_2_O	110	330.0	—

Abbreviations: Fe, iron; FeSO_4_, ferrous sulfate; H_2_O, water; NaFeEDTA, sodium iron ethylenediaminetetraacetate.

### INFOGEST procedure

Initially, electrolyte solutions, simulated salivary fluid, simulated gastric fluid, and simulated intestinal fluid (SIF) were prepared and used for the digestion phases [9]. Manufacturers recommend reassessing enzyme activities when the products are stored for >3 y. As all reagents used in this study were newly purchased and stored for <1 y, the enzyme activity values provided by manufacturer (Sigma Aldrich) were utilized directly. In the oral phase, 5 g of food samples were mixed with 4 mL of simulated salivary fluid and 1 mL of amylase solution (83 mg amylase per milliliter water, 9 enzymatic activity units per milligram amylase) in 50 mL centrifuge tubes. The oral boluses were manually minced with stainless steel spatulas and incubated at 37°C for 2 min. The oral boluses were added to 8 mL of simulated gastric fluid in the gastric phase. After the pH values were reduced to 3 ± 0.1 with 6 M HCl, 1 mL of pepsin solution (66 mg pepsin per milliliter water, 605 enzymatic activity units per milligram pepsin) and 1 mL of lipase solution (80 mg lipase per milliliter water, 15 enzymatic activity units per milligram lipase) were added and placed on a shaking incubator plate (211DS, Labnet International, Inc.) at 150 × *g*, 2 h, 37°C. In the intestinal phase, 8 mL of SIF was mixed with the gastric chyme, and then pH values were adjusted to 7 ± 0.1 with 5 M sodium hydroxide. Three milliliters of bile solution (54 mg bile per milliliter SIF, 10 mM), 5 mL of pancreatin solution (4 mg pancreatin per milliliter SIF, 200 enzymatic activity units per milligram pancreatin), and 4 mL of water were added and incubated at 150 × *g*, 2 h, 37°C. Digesta was heated in the boiling water at 100°C for 5 min to stop intestinal digestion before being centrifuged at 5000 × *g*, 45 min, 5°C. Supernatant was collected using the 10 mL syringe with a 25 mm needle (18-gauge, 1 inch) without disturbing the chyme mass at the bottom of the tube. The iron content of dry samples and supernatant was analyzed with inductively coupled plasma-optical emission spectrometry (AOAC International Official Method 993.14: Trace Elements in Waters and Wastewaters) method by the Soil Testing laboratory at Kansas State University. Solubility (percentage) = 100 × soluble iron content (supernatant)/total iron content (dry sample) [9,[30], [31], [32], [33]].

**TABLE 2:** Experiment 1 Iron solubility.

Sample identification	Fe component	Fe solubility (%)
1	Fumarate	6.7 ± 0.28^b^
2	NaFeEDTA	20.2 ± 0.82^a^
3	FeSO_4_	7.3 ± 0.04^b^

Values are presented in mean ± SEM, *n* = 3. Values with different letters were significantly different (*P* < 0.05).

Abbreviations: Fe, iron; FeSO_4_, ferrous sulfate; NaFeEDTA, sodium iron ethylenediaminetetraacetate.

### Preparation of 3 iron treatment systems

#### Glahn’s dual-chamber system

**FIGURE 1:**
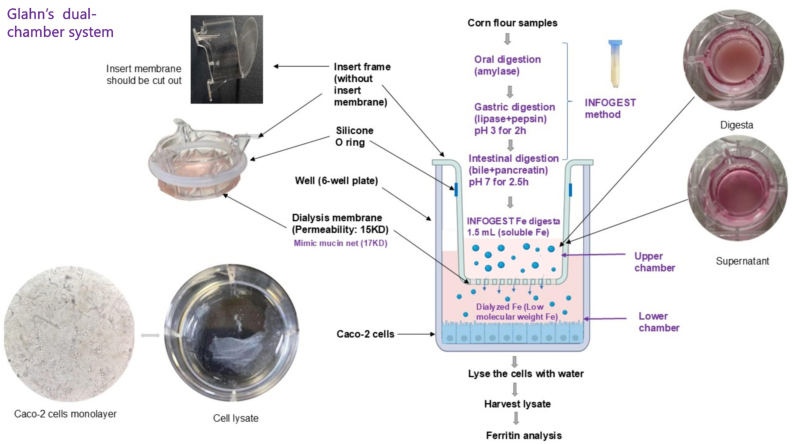
Glahn’s dual-chamber system with insert frame and dialysis membrane. Fe, iron.

The insert membrane was cut out with scissors. The piece of dialysis membrane (15 kDa, 1‒2 nm, regenerated cellulose) was held by the silicone O-ring at the bottom of insert frame. The well was separated by the dialysis membrane-insert system into the upper chamber and lower chamber (Figure 1) [4].

#### Alternative dual-chamber system

**FIGURE 2:**
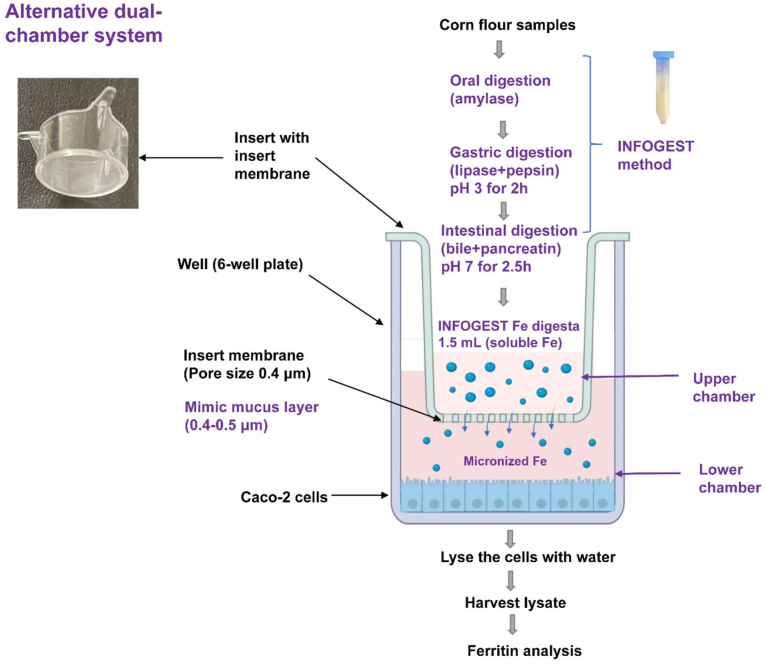
Alternative dual-chamber system with insert membrane. Fe, iron.

The insert membrane (0.4 *μ*m, polyethylene terephthalate) instead of the dialysis membrane was used (Figure 2).

#### Triple-chamber system

**FIGURE 3:**
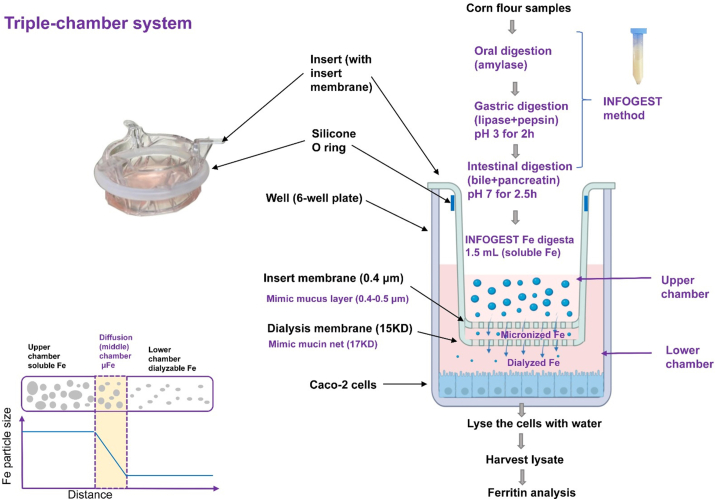
Triple-chamber system with insert membrane and dialysis membrane. Fe, iron.

The insert and dialysis membranes were used together. The piece of dialysis membrane (15 Molecular Weight Cut-Off) was held by the silicone O-ring at the bottom of the insert membrane. The 2 membranes separated the well into 3 compartments: upper chamber, middle chamber, and lower chamber. The height of the middle chamber was ∼1 mm (Figure 3).

### Caco-2 cell culture procedure

Caco-2 cells were purchased from American Type Culture Collection at passage 18. The Caco-2 cells were cultured in T25, T75, and T225 flasks with Dulbecco’s modified Eagle’s medium (DMEM) supplemented with 10% fetal bovine serum, 1% ZellShield, and 25 mM 4-(2-hydroxyethyl)-1-piperazineethanesulfonic acid (HEPES) and incubated at 37°C in 5% carbon dioxide air [4].

Caco-2 cells were detached with trypsin (0.25%)-EDTA (0.53 mM) for 5‒10 min at 80%‒100% confluency. Dissociated cells were centrifuged at 125 × *g* for 5‒10 min. Cells were counted with a hemocytometer. Caco-2 cells (passage 23) from T225 flasks were transferred to collagen-coated 6-well plates with 2 mL of DMEM per well at a seeding density of 50,000 cells/cm^2^ and allowed to grow for 12 d with media being refreshed every 2 d [4].

On the twelfth day, DMEM was replaced by 2 mL of minimum essential medium (MEM) supplemented with 4 mg/L hydrocortisone, 10 mM Piperazine-1,4-bis (2-ethanesulfonic acid) disodium salt, 20 μg/L epidermal growth factor, 5 μg/L selenium, 34 μg/L triiodothyronine, 5 mg/L insulin, and 1% ZellShield [4]. Iron digesta or supernatant was produced using INFOGEST method and stored overnight at ‒80°C [33].

On the thirteenth day, insert frames, silicone O-ring, and dialysis membranes were prepared and sterilized with 0.5 M HCl solution for ≥1 h, and then rinsed with autoclaved deionized water. Two milliliters of MEM were removed. One milliliter of fresh MEM was added to the lower chamber, and 1.5 mL of iron digesta or supernatant was added to the upper chamber. Iron treatments were randomly assigned to Caco-2 cells by a table generated with Microsoft Excel. The 6-well plates were placed on a rocking shaker [6 oscillations/min, VWR (Avantor)] in the incubator (Forma Scientific, Inc.) for 2 h. The dual- or triple- chamber system was removed, and 1 mL of MEM was added. The Caco-2 cells were returned to the incubator for another 22 h. A control (only media) was used as a reference.

On the fourteenth day, MEM was removed, and autoclaved deionized water was added to osmotically lyse the Caco-2 cells [4]. The 6-well plate was put on the tube rack in the sonicator (Branson 2510) for 30 s to further lyse cells [34]. Finally, cell lysate was collected for ferritin and protein analyses according to the instructions of ferritin and protein kits [4]. Ferritin values presented are not background-subtracted. We conducted an additional iron fortification experiment evaluating FePP and Ferric orthophosphate FePO_4_ in a companion study, using the INFOGEST digestion method and the Caco-2 cell model with Glahn’s dual-chamber system [35].

### Statistical analysis

Data were analyzed in SAS online studio (https://welcome.oda.sas.com/) with significance at *P* value < 0.05. The Shapiro-Wilk test was used for normality, and Levene’s test for homogeneity. Data were analyzed by 1-way analysis of variance with the least significant difference test. ELISA analysis was performed using the Arigo online calculator (regression model) at https://www.arigobio.com/elisa-analysis.

## Results

### Supernatant iron solubility

In experiment 1, NaFeEDTA iron solubility was significantly higher than ferrous fumarate and FeSO_4_ (Table 2). In experiment 2, the combinations of NaFeEDTA and ferrous fumarate/FeSO_4_ at a 4:9 weight ratio had significantly higher iron solubility than the combinations of NaFeEDTA and ferrous fumarate/FeSO_4_ at a 2:11 weight ratio (Table 3).

**TABLE 3:** Experiment 2 Iron solubility.

Sample identification	Fe component	Weight ratio	Fe solubility (%)
4	NaFeEDTA + fumarate	4:9	13.3 ± 0.75^a^
5	NaFeEDTA + FeSO_4_	4:9	15.0 ± 0.69^a^
6	NaFeEDTA + fumarate	2:11	10.1 ± 0.48^b^
7	NaFeEDTA + FeSO_4_	2:11	9.1 ± 0.54^b^

Values are presented in mean ± SEM, *n* = 3. Values with different letters were significantly different (*P* < 0.05).

Abbreviations: Fe, iron; FeSO_4_, ferrous sulfate; NaFeEDTA, sodium iron ethylenediaminetetraacetate.

**TABLE 4:** Experiment 1 Ferritin concentrations.

Sample identification	Fe component	Fe additive form	Fe treatment system	Fe bioavailability (ferritin concentration ng/mg protein)	Total cell protein content (mg/well)
1	Fumarate	Digesta	Glahn’s dual-chamber	110.21 ± 9.08^ab^	0.96 ± 0.04
2	NaFeEDTA	Digesta	Glahn’s dual-chamber	119.37 ± 3.05^a^	0.53 ± 0.08
3	FeSO_4_	Digesta	Glahn’s dual-chamber	96.52 ± 9.09^bc^	0.69 ± 0.08
1	Fumarate	Supernatant	Glahn’s dual-chamber	79.30 ± 12.04^cd^	0.80 ± 0.12
2	NaFeEDTA	Supernatant	Glahn’s dual-chamber	68.43 ± 19.78^d^	1.06 ± 0.15
3	FeSO_4_	Supernatant	Glahn’s dual-chamber	90.51 ± 9.79^c^	0.81 ± 0.05
1	Fumarate	Digesta	Alternative dual-chamber	6.10 ± 1.29^e^	0.74 ± 0.03
2	NaFeEDTA	Digesta	Alternative dual-chamber	4.38 ± 0.70^e^	0.69 ± 0.02
3	FeSO_4_	Digesta	Alternative dual-chamber	3.05 ± 0.38^e^	0.51 ± 0.04
1	Fumarate	Supernatant	Alternative dual-chamber	5.49 ± 1.07^e^	0.44 ± 0.02
2	NaFeEDTA	Supernatant	Alternative dual-chamber	4.50 ± 0.90^e^	0.76 ± 0.20
3	FeSO_4_	Supernatant	Alternative dual-chamber	5.39 ± 1.01^e^	0.71 ± 0.06
1	Fumarate	Digesta	Triple-chamber	3.60 ± 0.80^e^	0.68 ± 0.08
2	NaFeEDTA	Digesta	Triple-chamber	5.73 ± 1.81^e^	0.57 ± 0.08
3	FeSO_4_	Digesta	Triple-chamber	4.57 ± 0.43^e^	0.95 ± 0.05
1	Fumarate	Supernatant	Triple-chamber	2.82 ± 0.74^e^	0.91 ± 0.12
2	NaFeEDTA	Supernatant	Triple-chamber	3.49 ± 0.51^e^	0.73 ± 0.08
3	FeSO_4_	Supernatant	Triple-chamber	3.63 ± 0.41^e^	0.77 ± 0.04
Control	None	None	None	1.67 ± 0.3^e^	0.93 ± 0.07

Value presented in mean ± SEM, *n* = 3. Values with different letters were significantly different (*P* < 0.05).

Abbreviations: Fe, iron; FeSO_4_, ferrous sulfate; NaFeEDTA, sodium iron ethylenediaminetetraacetate.

### Cellular iron bioavailability

In experiment 1, NaFeEDTA, ferrous fumarate, and FeSO_4_ digesta and supernatant ferritin concentration in Glahn’s dual-chamber system were significantly higher than those of NaFeEDTA, ferrous fumarate, and FeSO_4_ in the alternative dual-chamber system and the triple-chamber system, respectively (Table 4). In Glahn’s dual-chamber system, NaFeEDTA digesta and ferrous fumarate digesta had significantly higher ferritin concentrations than NaFeEDTA supernatant and ferrous fumarate supernatant. There were no differences in ferritin concentration between FeSO_4_ digesta and FeSO_4_ supernatant. In Glahn’s dual-chamber system, the ferritin concentrations of ferrous fumarate and NaFeEDTA digesta were significantly higher than those of FeSO_4_ digesta, whereas the ferritin concentration of FeSO_4_ supernatant was significantly higher than that of NaFeEDTA supernatant (Table 4). Since Glahn’s dual-chamber system using digesta led to the highest ferritin concentrations, it was selected to use for experiment 2. In experiment 2, the combination of NaFeEDTA and FeSO_4_ at a 2:11 weight ratio had significantly higher ferritin concentration than the other combination samples (Table 5). The control (only media) is included in Table 5 as a reference.

**TABLE 5:** Experiment 2 Ferritin concentrations.

Sample identification	Fe component	Fe additive form	Fe treatment system	Fe bioavailability (ferritin concentration ng/mg protein)	Total cell protein content (mg/well)
4	NaFeEDTA + fumarate (4:9)	Digesta	Glahn’s dual-chamber	48.05 ± 8.95^b^	0.38 ± 0.03
5	NaFeEDTA + FeSO_4_ (4:9)	Digesta	Glahn’s dual-chamber	58.87 ± 13.17^b^	0.48 ± 0.03
6	NaFeEDTA + fumarate (2:11)	Digesta	Glahn’s dual-chamber	56.99 ± 5.92^b^	0.51 ± 0.02
7	NaFeEDTA + FeSO_4_ (2:11)	Digesta	Glahn’s dual-chamber	96.12 ± 5.24^a^	0.42 ± 0.05
Control	—	—	—	3.91 ± 0.67^c^	0.36 ± 0.02

Values are presented in mean ± SEM, *n* = 3. Values with different letters were significantly different (*P* < 0.05).

Abbreviations: Fe, iron; FeSO_4_, ferrous sulfate; NaFeEDTA, sodium iron ethylenediaminetetraacetate.

## Discussion

The alternative dual-chamber system and the triple-chamber system resulted in ferritin concentrations that were not significantly different from the control. One possible explanation is that the soluble iron did not diffuse into the lower chamber despite the larger molecular weight (0.4 μm), or it bound to the membrane. It may be because the material of the insert membrane (polyethylene terephthalate) does not have good permeability for this type of application. It is also possible that cell function was compromised by enzymes and bile salts reaching them through the membrane. Another possible explanation is that, compared with the 0.4 μm membrane insert, the 8 μm membrane insert may serve as a better substitute for the 15 kDa dialysis membrane [24]. Bering et al. [36] also designed a triple-chamber system. However, the necessity and reliability of their triple-chamber system were questioned [15,36]. In the present study, the Caco-2 cell model using Glahn’s dual-chamber system was the most effective bioassay. Consistent with these findings it has been used previously to evaluate the iron bioavailability of milk fortified with NaFeEDTA, ferrous fumarate, or FeSO_4_ [37]. There was no difference in iron bioavailability of NaFeEDTA compared with ferrous fumarate and FeSO_4_, but NaFeEDTA exhibited significantly higher dialyzed iron concentrations than ferrous fumarate and FeSO_4_ [37].

To our surprise, despite the higher INFOGEST digesta content, the ferritin concentrations did not seem to indicate that ferritin concentration was saturated, since NaFeEDTA, ferrous fumarate, and FeSO_4_ were superior or equivalent to their supernatant in ferritin response. It may be that not all the soluble iron was separated from chyme mass in the supernatant by centrifuging. This is a possible explanation for the poor predictive ability of iron solubility for iron bioavailability [2,38]. Therefore, many prefer to expose Caco-2 cells to digesta rather than supernatant [[39], [40], [41]], since the digesta contains not only soluble nutrients but also other small complexes and digestive products derived from food components (e.g., micellar particles, colloidal particles, monosaccharides, oligosaccharides, short peptides, monoglycerides, and free fatty acids) [42]. Digesta more closely resembles the intestinal chyme and provides a more physiologically relevant environment for assessing nutrient bioavailability.

We found that all combinations were effective (48.1‒96.1 ng ferritin per milligram protein). Surprisingly, the combination of NaFeEDTA and FeSO_4_ at a 2:11 weight ratio had a significantly higher ferritin response. When FeSO_4_ is dissolved in water, it can form an aquo complex [Fe(H_2_O)_6_]^2+^ [43]. One possible reason is that EDTA can react with [Fe(H_2_O)_6_]^2+^ to maximize the iron absorption at a specific ratio. Some scientists found that isotopic iron absorption could be elevated to 5.7% and 2.9% at EDTA-Fe (FeSO_4_) molar ratios of 0.67:1 and 1:1 [44]. In children, ^57^FeSO_4_-fortified meals with EDTA-Fe molar ratios of 0.4:1 or 0.7:1 were tested. Only the meal with a ratio of 0.4:1 significantly increased the absorption rate of isotopic iron [45]. Their results are consistent with our finding that the combination of NaFeEDTA and FeSO_4_ with low weight ratio (2:11) had higher iron bioavailability than the combination of NaFeEDTA and FeSO_4_ with high weight ratio (4:9). In a clinical trial, ^57/58^Fe absorption significantly improved to 3.7%, 3.5%, and 3.8% at EDTA-Fe (FeSO_4_) molar ratios of 1:1, 0.7:1, and 0.3:1, compared with control meals without enhancers [46]. In contrast, another clinical trial on radio-labeled EDTA-FeSO_4_ fortifications, the results of iron absorption were 11.3%, 13.5%, and 8.8% at EDTA-Fe (FeSO_4_) molar ratios of 0.25:1, 0.5:1, and 1:1 [47]. Thus, it seems that there may be a curvilinear relationship depending on various influencing factors. More research is needed to better understand the relationship between weight ratios of EDTA-FeSO_4_ and iron bioavailability.

Several limitations should be noted from this work. The total well protein (0.36‒1.1 mg) concentrations were lower than the expected range (2.3‒2.7 mg). However, baseline ferritin concentrations (1.7‒3.9 ng ferritin per milligram cell protein) were in the expected range (2–4 ng ferritin per milligram cell protein). This might result from cell protein coagulation during cell collection and analysis (Figure 1). Protein and ferritin concentrations might appear relatively higher when thick cell lysates were analyzed, and lower when thin cell lysates were analyzed. However, the ferritin-to-protein ratios would remain unaffected. Cell viability and membrane integrity were also not assessed in this study. In addition, a blank containing only deionized water (without cells) in the lower chamber was not established to determine the concentration of soluble/dialysis iron on the cell side after 2 h of iron treatment.

The commercial corn flour contained 4.67 mg Fe/100 g corn. It is possible that commercial corn flour had been subject to some degree of iron fortification, and/or iron contamination. However, this limitation did not affect the iron fortification concentration used in these studies. Finally, the heat-shock step (100°C for 5 min) may impact the iron complexes, promoters, and inhibitors. This can be considered in the context of extrusion. In typical extrusion processes, barrel temperatures range from 40°C to 90°C, with peak temperature reaching 104°C‒110°C. The residence time at peak temperatures is longer than 5 min. Previous studies on iron-fortified extruded rice flour reported inconsistent findings. Some observed that extrusion could significantly improve iron solubility and bioavailability [8], whereas others indicated that extrusion did not significantly affect iron bioavailability [7]. Therefore, the available evidence does not support a clear consensus on how this heat-shock affects iron bioavailability. It is worth noting that we have investigated rice iron fortification outcomes using this model and rats [].

In conclusion, the Caco-2 cell model with Glahn’s dual-chamber system and INFOGEST digesta is a reliable iron bioassay. NaFeEDTA had higher bioavailability than FeSO_4,_ but not ferrous fumarate. The combination of NaFeEDTA and FeSO_4_ at a 2:11 weight ratio has the highest bioavailability among the combination formulations.

## Author contributions

The authors’ responsibilities were as follows – JZ: conducted the cell culture study and wrote the paper; JZ, BLL: revised the paper; BLL: had primary responsibility for the final content. Both authors read and approved the final manuscript.

## Data availability

The authors confirm that all data supporting the findings of this study are included within the manuscript. SAS code book and analytic code are freely and publicly accessible without restriction at https://support.sas.com/documentation/onlinedoc/stat/131/anova.pdf and https://video.sas.com/detail/video/5537529513001/the-one-way-anova-task-in-sas-studio

## Declaration of generative AI and AI-assisted technologies in the writing process

The authors declare that no generative AI or AI-assisted technologies were used in the writing of this manuscript.

## Funding

This work was supported by USDA Multistate 4002 and the Kansas Agricultural Experiment Station.

## Conflict of interest

The authors report no conflicts of interest.

## References

[1] Miller D.D., Berner L.A. (1989). Is solubility in vitro a reliable predictor of iron bioavailability?. Biol. Trace Elem. Res..

[2] Pynaert I., Armah C., Fairweather-Tait S., Kolsteren P., van Camp J., De Henauw S.D. (2006). Iron solubility compared with in vitro digestion–Caco-2 cell culture method for the assessment of iron bioavailability in a processed and unprocessed complementary food for Tanzanian infants (6-12 months). Br. J. Nutr..

[3] Engle-Stone R., Yeung A., Welch R., Glahn R. (2005). Meat and ascorbic acid can promote Fe availability from Fe-phytate but not from Fe-tannic acid complexes. J. Agric. Food Chem..

[4] Glahn R.P. (2022). The caco-2 cell bioassay for measurement of food iron bioavailability. J. Vis. Exp..

[5] Mamiro P.S., Kolsteren P.W., van Camp J.H., Roberfroid D.A., Tatala S., Opsomer A.S. (2004). Processed complementary food does not improve growth or hemoglobin status of rural Tanzanian infants from 6-12 months of age in Kilosa district, Tanzania. J. Nutr..

[6] Hackl L., Zimmermann M.B., Zeder C., Parker M., Johns P.W., Hurrell R.F. (2017). Iron bioavailability from ferric pyrophosphate in extruded rice Cofortified with zinc sulfate is greater than when Cofortified with zinc oxide in a human stable isotope study. J. Nutr..

[7] Scheuchzer P., Zimmerman M.B., Zeder C., Sánchez-Ferrer A., Moretti D. (2022). Higher extrusion temperature induces Greater Formation of less digestible Type V and retrograded starch in iron-fortified rice grains but does not affect iron bioavailability: stable isotope studies in young women. J. Nutr..

[8] Scheuchzer P., Syryamina V.N., Zimmermann M.B., Zeder C., Nyström L., Yulikov M. (2023). Ferric pyrophosphate forms soluble iron coordination complexes with zinc compounds and solubilizing agents in extruded rice and predicts increased iron solubility and bioavailability in young women. J. Nutr..

[9] Brodkorb A., Egger L., Alminger M., Alvito P., Assunção R., Ballance S. (2019). INFOGEST static in vitro simulation of gastrointestinal food digestion. Nat. Protoc..

[10] da Silva Menezes M.C., Miyahira R.F., Pimenta Inada K.O., Fonseca dos Santos Y., da Costa Marques Calderari M.R., Beres C. (2025). Adverse influence of chia seed germination on iron bioaccessibility and uptake by Caco-2 cells. Food Biosci.

[11] Oliveira A.S., Ferreira C.M., Pereira J.O., Silva S., Costa E.M., Pereira A.M. (2023). Iron-peptide complexes from spent yeast: evaluation of iron absorption using a Caco-2 monolayer. Food Biosci.

[12] Rebellato A.P., da Paixão Teixeira J.L., Milani R.F., Alvim I.D., da Silva Santana T.M., Galland F. (2025). Ferrous sulfate microparticles as a food fortification strategy: application in plant-based yogurt and bioaccessibility assessment. Food Res. Int..

[13] Nascimento P.A., Menezes I.M., Confortin C., Micheletto J., Filipak Neto F., Oliveira Ribeiro C.A. (2024). Bioaccessibility and bioavailability of essential and potentially toxic trace elements in potato cultivars: a comprehensive nutritional evaluation. Food Res Int.

[14] Stefos G.C., Dalaka E., Papoutsi G., Palamidi I., Andreou V., Katsaros G. (2024). In vitro evaluation of the effect of yogurt acid whey fractions on iron bioavailability. J. Dairy Sci..

[15] Fairweather-Tait S., Phillips I., Wortley G., Harvey L., Glahn R.P. (2007). The use of solubility, dialyzability, and Caco-2 cell methods to predict iron bioavailability. Int. J. Vitam. Nutr. Res..

[16] Birchenough G.M., Johansson M.E., Gustafsson J.K., Bergström J.H., Hansson G.C. (2015). New developments in goblet cell mucus secretion and function, Mucosal. Immunol.

[17] Nilsson H.E., Ambort D., Bäckström M., Thomsson E., Koeck P.J., Hansson G.C. (2014). Intestinal MUC2 mucin supramolecular topology by packing and release resting on D3 domain assembly. J. Mol. Biol..

[18] Paone P., Cani P.D. (2020). Mucus barrier, mucins and gut microbiota: the expected slimy partners?. Gut.

[19] Herath M., Hosie S., Bornstein J.C., Franks A.E., Hill-Yardin E.L. (2020). The role of the gastrointestinal mucus system in intestinal homeostasis: implications for neurological disorders. Front Cell Infect Microbiol.

[20] McCright J., Sinha A., Maisel K. (2022). Generating an in vitro gut model with physiologically relevant biophysical mucus properties. Cell Mol. Bioeng..

[21] Leal J., Smyth H.D., Ghosh D. (2017). Physicochemical properties of mucus and their impact on transmucosal drug delivery. Int. J. Pharm..

[22] Mahler G.J., Shuler M.L., Glahn R.P. (2009). Characterization of Caco-2 and HT29-MTX cocultures in an in vitro digestion/cell culture model used to predict iron bioavailability. J. Nutr. Biochem..

[23] Laparra J.M., Glahn R.P., Miller D.D. (2009). Different responses of Fe transporters in Caco-2/HT29-MTX cocultures than in independent Caco-2 cell cultures. Cell Biol. Int..

[24] Jin F., Welch R., Glahn R. (2006). Moving toward a more physiological model: application of mucin to refine the in vitro digestion/Caco-2 cell culture system. J. Agric. Food Chem..

[25] Zhu L., Yeung C.K., Glahn R.P., Miller D.D. (2006). Iron dissociates from the NaFeEDTA complex prior to or during intestinal absorption in rats. J. Agric Food Chem..

[26] Bothwell T.H., MacPhail A.P. (2004). The potential role of NaFeEDTA as an iron fortificant. Int. J. Vitam. Nutr. Res..

[27] Webb P., Rogers B.L., Rosenberg I., Schlossman N., Wanke C., Bagriansky J. (2011). https://citeseerx.ist.psu.edu/document?repid=rep1%26type=pdf%26doi=51947bcdd7f09a0fe6fa4caa0f8680c77060912f.

[28] Delimont N.M., Vahl C.I., Kayanda R., Msuya W., Mulford M., Alberghine P. (2019). Complementary feeding of sorghum-based and corn-based fortified blended foods results in similar iron, vitamin A, and anthropometric outcomes in the MFFAPP Tanzania efficacy study. Curr. Dev. Nutr..

[29] Walter T., Pizarro F., Olivares M. (2003). Iron bioavailability in corn-masa tortillas is improved by the addition of disodium EDTA. J. Nutr..

[30] Muleya M., Young S.D., Bailey E.H. (2021). A stable isotope approach to accurately determine iron and zinc bioaccessibility in cereals and legumes based on a modified INFOGEST static in vitro digestion method. Food Res. Int..

[31] Minekus M., Alminger M., Alvito P., Ballance S., Bohn T., Bourlieu C. (2014). A standardised static in vitro digestion method suitable for food‒an international consensus. Food Funct.

[32] Hackl L., Cercamondi C.I., Zeder C., Wild D., Adelmann H., Zimmermann M.B. (2016). Cofortification of ferric pyrophosphate and citric acid/trisodium citrate into extruded rice grains doubles iron bioavailability through in situ generation of soluble ferric pyrophosphate citrate complexes. Am. J. Clin. Nutr..

[33] Penugonda K., Fiorentino N.M., Alavi S., Lindshield B.L. (2018). Bioavailable iron and vitamin A in newly formulated, extruded corn, soybean, sorghum, and cowpea fortified-blended foods in the in vitro digestion/Caco-2 cell model. Curr. Dev. Nutr..

[34] Webb Patrick, Lorge Rogers Beatrice, Irwin Rosenberg, Schlossman Nina, Christine Wanke, Jack Bagriansky, Kate Sadler, Johnson Quentin, Jessica Tilahun, Amelia Reese Masterson (2011). Improving the Nutritional Quality of U.S. Food Aid: Recommendations for Changes to Products and Programs.

[35] J. Zhang, D.B. Akinbo, E.J. Ward, H.R. Suleria, T. Graff, M. Joseph et al., Bioavailability of Ferric Pyrophosphate and Ferric Orthophosphate with/without Extrusion and/or Citric Acid and/or Trisodium Citrate in rats and in vitro INFOGEST Digestion and Caco-2 Cell Model, Current Developments in Nutrition, Under Review 10.1016/j.cdnut.2026.107718 PMC13263688 42291142

[36] Bering S., Bukhave K., Henriksen M., Sandström B., Pariagh S., Fairweather-Tait S.J. (2006). Development of a three-tier in vitro system, using Caco-2 cells, to assess the effects of lactate on iron uptake and transport from rye bread following in vitro digestion. J. Sci. Food Agric..

[37] Yeung A.C., Glahn R.P., Miller D.D. (2002). Comparison of the availability of various iron fortificants in bread and milk using an in vitro digestion/Caco-2 cell culture method. J. Food Sci..

[38] Glahn R.P., Wien E.M., Van Campen D.R., Miller D.D. (1996). Caco-2 cell iron uptake from meat and casein digests parallels in vivo studies: use of a novel in vitro method for rapid estimation of iron bioavailability. J. Nutr..

[39] Latunde-Dada G.O., Li X., Parodi A., Edwards C.H., Ellis P.R., Sharp P.A. (2014). Micromilling enhances iron bioaccessibility from wholegrain wheat. J. Agric. Food Chem..

[40] Lei J., Zhang Y., Chen X.G., Zhang M.Q., Bai L., Huang C.Y. (2012). Assessment of iron bioavailability in ten kinds of Chinese wheat flours using an in vitro digestion/Caco-2 cell model. Biomed. Environ. Sci..

[41] Pullakhandam R., Nair K.M., Pamini H., Punjal R. (2011). Bioavailability of iron and zinc from multiple micronutrient fortified beverage premixes in Caco-2 cell model. J. Food Sci..

[42] Lu W., Nishinari K., Phillips G.O., Fang Y. (2021). Colloidal nutrition science to understand food-body interaction. Trends Food Sci. Technol..

[43] (2015). Reactions of ions in aqueous solution.

[44] Hurrell R.F., Reddy M.B., Burri J., Cook J.D. (2000). An evaluation of EDTA compounds for iron fortification of cereal-based foods. Br. J. Nutr..

[45] Chang S., Huang Z., Ma Y., Piao J., Yang X., Zeder C. (2012). Mixture of ferric sodium ethylenediaminetetraacetate (NaFeEDTA) and ferrous sulfate: an effective iron fortificant for complementary foods for young Chinese children. Food Nutr. Bull..

[46] Davidsson L., Walczyk T., Zavaleta N., Hurrell R. (2001). Improving iron absorption from a Peruvian school breakfast meal by adding ascorbic acid or Na2EDTA. Am. J. Clin. Nutr..

[47] MacPhail A.P., Patel R.C., Bothwell T.H., Lamparelli R.D. (1994). EDTA and the absorption of iron from food. Am. J. Clin. Nutr..

